# Ultraflexible PEDOT:PSS/IrO_x_-Modified Electrodes: Applications in Behavioral Modulation and Neural Signal Recording in Mice

**DOI:** 10.3390/mi15040447

**Published:** 2024-03-27

**Authors:** Xueying Wang, Wanqi Jiang, Huiran Yang, Yifei Ye, Zhitao Zhou, Liuyang Sun, Yanyan Nie, Tiger H. Tao, Xiaoling Wei

**Affiliations:** 1 State Key Laboratory of Transducer Technology, Shanghai Institute of Microsystem and Information Technology, Chinese Academy of Sciences, Shanghai 200050, China; voyteywang@mail.sim.ac.cn (X.W.); jiangwanqi@mail.sim.ac.cn (W.J.); hryang@mail.sim.ac.cn (H.Y.); yeyifei@mail.sim.ac.cn (Y.Y.); ztzhou@mail.sim.ac.cn (Z.Z.); liuyang.sun@mail.sim.ac.cn (L.S.); tiger@mail.sim.ac.cn (T.H.T.); 2 School of Graduate Study, University of Chinese Academy of Sciences, Beijing 100049, China; 3 2020 X-Lab, Shanghai Institute of Microsystem and Information Technology, Chinese Academy of Sciences, Shanghai 200050, China; 4 Shanghai Laboratory Animal Research Center, Shanghai 201203, China; neiyanyan@slarc.org.cn; 5 School of Physical Science and Technology, ShanghaiTech University, Shanghai 201210, China; 6 Center of Materials Science and Optoelectronics Engineering, University of Chinese Academy of Sciences, Beijing 100049, China; 7 Center for Excellence in Brain Science and Intelligence Technology, Chinese Academy of Sciences, Shanghai 200031, China; 8 Neuroxess Co., Ltd. (Jiangxi), Nanchang 330029, China; 9 Guangdong Institute of Intelligence Science and Technology, Hengqin, Zhuhai 519031, China; 10 Tianqiao and Chrissy Chen Institute for Translational Research, Shanghai 200040, China

**Keywords:** ultraflexible probe, intracortical, PEDOT:PSS/IrO_x_ modification, bilateral BCI, behavior modulation

## Abstract

Recent advancements in neural probe technology have become pivotal in both neuroscience research and the clinical management of neurological disorders. State-of-the-art developments have led to the advent of multichannel, high-density bidirectional neural interfaces that are adept at both recording and modulating neuronal activity within the central nervous system. Despite this progress, extant bidirectional probes designed for simultaneous recording and stimulation are beset with limitations, including elicitation of inflammatory responses and insufficient charge injection capacity. In this paper, we delineate the design and application of an innovative ultraflexible bidirectional neural probe engineered from polyimide. This probe is distinguished by its ability to facilitate high-resolution recordings and precise stimulation control in deep brain regions. Electrodes enhanced with a PEDOT:PSS/IrO_x_ composite exhibit a substantial increase in charge storage capacity, escalating from 0.14 ± 0.01 mC/cm^2^ to an impressive 24.75 ± 0.18 mC/cm^2^. This augmentation significantly bolsters the electrodes’ charge transfer efficacy. In tandem, we observed a notable reduction in electrode impedance, from 3.47 ± 1.77 MΩ to a mere 41.88 ± 4.04 kΩ, while the phase angle exhibited a positive shift from −72.61 ± 1.84° to −34.17 ± 0.42°. To substantiate the electrodes’ functional prowess, we conducted in vivo experiments, where the probes were surgically implanted into the bilateral motor cortex of mice. These experiments involved the synchronous recording and meticulous analysis of neural signal fluctuations during stimulation and an assessment of the probes’ proficiency in modulating directional turning behaviors in the subjects. The empirical evidence corroborates that targeted stimulation within the bilateral motor cortex of mice can modulate the intensity of neural signals in the stimulated locale, enabling the directional control of the mice’s turning behavior to the contralateral side of the stimulation site.

## 1. Introduction

With the advancement of fabrication technologies, a diverse array of neural interfaces has undergone extensive research and development. Owing to the superior signal quality and resolution offered by deep brain electrodes, implantable deep brain probes have attracted considerable attention and investigation. Serving as devices that can both monitor and stimulate the nervous system, deep neural interfaces have been widely applied in neuroscientific research, such as mapping neural circuits, as well as in the treatment of neurodegenerative diseases like Parkinson’s disease, epilepsy, and amyotrophic lateral sclerosis (ALS) [1,2]. Deep neural probes can capture high-resolution local field potentials (LFPs) and discern the firing activity of individual neurons [3]. Additionally, they facilitate the development of in situ capabilities of stimulations [4]. Bidirectional neural interfaces, which integrate recording and stimulation functionalities, not only achieve high-resolution recording of individual neuron activity but also allow for real-time modulation. This dual capability underscores their immense potential within the realm of neuroscience research.

Currently, the materials used for probe fabrication include traditional metal wires, silicon-based materials, and polymers. However, traditional metal wires and silicon materials exhibit a significant mechanical mismatch with brain tissue [5,6]. The Young’s modulus of silicon and metal is ten thousand times greater than that of brain tissue. Additionally, the slight movements of the brain within the skull during physiological activities such as respiration result in continuous cutting of the brain tissue by rigid probes during long-term implantation. This leads to persistent inflammation reactions in the brain tissue, causing the rigid probes to be encapsulated by severe glial scarring. The formation of glial scars severely affects the contact between the electrode and the surrounding neural tissue, gradually leading to electrode failure and hindering the long-term recording and stimulation performance of the electrode within the body [5,6,7].

In recent years, the development and advancements in research and processing techniques of flexible polymers have led to their increasing application in the development of neural interfaces, offering the potential for long-term in vivo neural signal recording and stimulation performance. Various polymer materials have already been applied in the development of neural interfaces, including SU8, Parylene, polyimide (PI), and polydimethylsiloxane (PDMS) [8,9,10,11,12,13]. These materials minimize the mechanical mismatch between the brain and flexible probes and reduce post-implantation displacement due to their lower Young’s modulus and smaller cross-sectional area, resulting in a smaller bending modulus. Owing to their flexibility, these probes can move with the brain’s micromotion. In addition to improved mechanical properties, these flexible polymers also exhibit excellent corrosion resistance, further enhancing their potential for long-term in-body performance. In terms of probe fabrication, combining Micro-Electro-Mechanical Systems (MEMS) surface processing techniques, these materials undergo similar processing techniques to silicon materials. Through processes such as photolithography, etching, and metallization, they achieve micrometer-level precision in fabrication, resulting in smaller dimensions and cross-sectional areas to minimize tissue damage upon insertion. This contributes to the improvement in neural recording and stimulation performance [9,12].

Although miniaturized and flexible neural interfaces are considered necessary for improving the long-term stability and spatial resolution of neural signal recording and stimulation, one challenge faced by these new microelectrodes is their higher impedance due to the smaller electrode–tissue interface. This high impedance can degrade signal quality, resulting in a lower signal-to-noise ratio (SNR). To address this issue, one approach is to modify the microelectrodes with nanoparticles such as platinum nanoparticles (PtNPs), poly(3,4-ethylenedioxythiophene):poly(styrene sulfonate) (PEDOT:PSS), and other materials to increase surface roughness and thereby enlarge the effective surface area of the electrode, reducing impedance [14,15]. PEDOT:PSS, as a conductive polymer, the application of this material can markedly decrease the impedance of microelectrodes spanning a broad frequency range while maintaining electrochemical stability [16]. This characteristic is instrumental in augmenting the fidelity of electrical recordings from the electrodes, ensuring a consistently stable performance. In terms of stimulation performance, PEDOT:PSS also shows great potential due to its high charge injection and storage capabilities [17,18]. Additionally, iridium oxide (IrO_x_) electrodes demonstrate higher charge storage capacity (CSC) as they undergo reversible Faradaic reactions between the Ir^3+^ and Ir^4+^ oxidation-reduction states in applied stimulation pulses [19], which further enhances their potential in the field of electrical stimulation.

In this study, we developed an ultraflexible recording–stimulating bidirectional neural interface. The electrode surface was modified using a composite modification method with PEDOT:PSS/IrO_x_, and the morphology and electrical characteristics of the electrodes under different modifications were characterized. The results showed that the PEDOT:PSS/IrO_x_ composite modification exhibits superior performance compared to the unmodified electrodes. It reduced the electrode impedance by 2 orders of magnitude and increased the CSC from 0.14 ± 0.01 mC/cm^2^ to 24.75 ± 0.18 mC/cm^2^, greatly improving the electrical recording and stimulation performance of the electrodes. We applied these electrodes to record and regulate the activity of neurons and movement of mice. The electrodes successfully recorded neural firing activities in the mouse motor cortex and allowed for the analysis of single neuron firing activity changes. By applying electrical stimulation to the bilateral secondary motor cortex (M2) brain region of the mice, we were able to control their left and right turning movements, successfully validating the functionality of the electrodes, and demonstrating their potential in the field of bidirectional neural interfaces.

## 2. Materials and Methods

### 2.1. Fabrication and Packaging of a Flexible Probe

**Figure 1:**
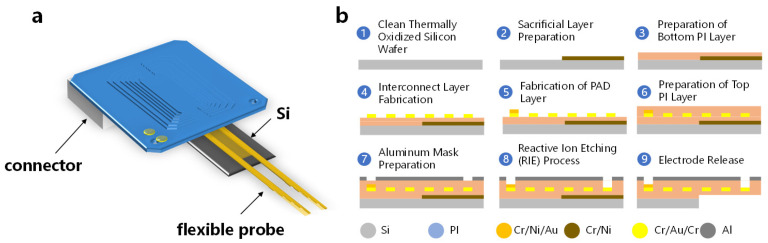
(**a**) Diagram of the flexible probe. (**b**) Schematic diagram of the flexible probe fabrication process.

**Figure 2:**
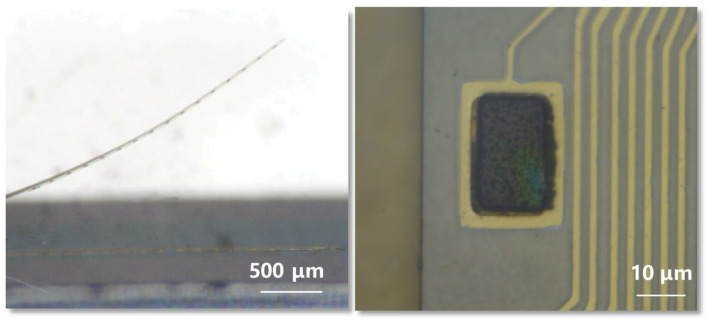
The front end of the flexible probe after release (**left**) and the modification electrode (**right**).

Probes were fabricated utilizing micro- and nanomanufacturing techniques tailored for four-inch wafers, coupled with packaging processes, as shown in Figure 1. The substrate for the flexible probe fabrication was a four-inch silicon wafer (n-type 0.005 V·cm, XiaMen LuYuan Science and Technology, Xiamen, China). To ensure optimal insulation, the wafer underwent thermal oxidation to develop a 2-micron-thick silicon dioxide insulating layer. The pattern for the sacrificial metal layer was delineated using photolithography and development processes, followed by the deposition of a 100 nm layer of nickel (Ni) on the patterned photoresist via electron beam evaporation. The final metal pattern emerged post the lift-off process. A polyimide layer (PI-2610, HD Microsystems, Parlin, NJ, USA) was then spin-coated to serve as the base insulating layer for the flexible probe and was cured under a nitrogen atmosphere at 380 °C. Subsequent photolithographic steps, electron beam evaporation, and the lift-off process were employed to form metal interconnects with a structure of Cr/Au/Cr with thicknesses of 5/150/5 nm and a width of 2 μm. Additionally, a metal pad with a diameter of 200 μm and a layered composition of Cr/Ni/Au with thicknesses of 5/100/50 nm was fabricated through photolithography, electron beam evaporation, and lift-off, serving as the under bump metallization (UBM) for subsequent soldering to the printed circuit board (PCB). Another polyimide layer was spin-coated atop as the upper insulating layer for the flexible probe. The flexible probe, electrode openings, and pads were then patterned using a photolithographic aluminum mask, exposing the underlying polyimide, which was subsequently removed through dry etching. This process revealed the metal underneath at the pad and electrode openings and defined the overall shape of the flexible probe, the front end of probe as shown in Figure 2.

Individual flexible probe devices were isolated through a dicing process. Subsequently, laser ball bonding technology was employed to create solder balls with a diameter of 150 μm on the device’s solder pads. These flexible probe devices were then affixed to a bespoke 32-channel PCB (Shenzhen Jialichuang Technology Group Co., Ltd., Shenzhen, China) via flip-chip bonding techniques. To fortify the mechanical robustness of the bonded connections, an epoxy resin was dispensed as underfill around the solder joints. These procedural steps culminated in the fabrication and packaging of the probe. The diagram of packaged probe is shown in Figure 1a.

### 2.2. The Modification Methods of Electrode

**Figure 3:**
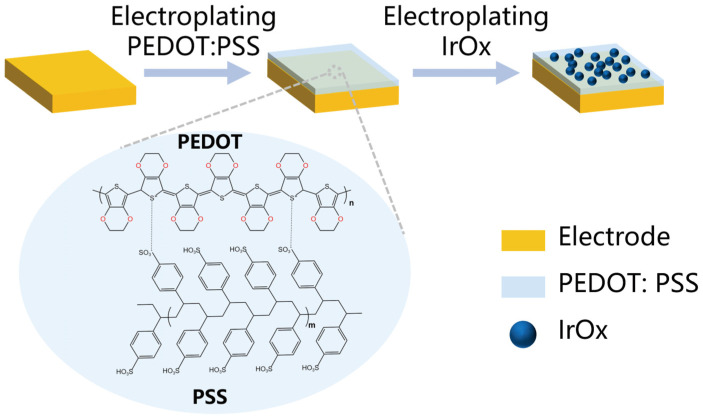
Schematic diagram of the electrode tip surface modification process.

Two different methods were employed to modify the electrodes, as shown in Figure 3. For the PEDOT:PSS modification, the Intan electroplating board (Intan Technologies, Los Angeles, CA, USA) was used in constant current mode. The electroplating solution consisted of 0.02 M PSS (Sigma-Aldrich, St. Louis, MO, USA) and 0.01 M EDOT (Sigma-Aldrich, USA). The electroplating parameters were set at +10 nA for 10 s. The Platinum (Pt) wire as the counter electrode.

As for IrO_x_ modification, the PSTrace4 (PalmSens BV, Houten, The Netherlands) electrochemical workstation was utilized for electroplating. A three-electrode system was employed, with the flexible probe serving as the working electrode, a Pt wire as the counter electrode, and a saturated calomel electrode (SCE) as the reference electrode. A cyclic voltammetry technique was employed, with a voltage range of 0.05 V to 0.55 V (vs. SCE) and a scan rate of 50 mV/s for 30 cycles.

Iridium chloride hydrate (H_2_Cl_6_Ir·xH_2_O, Sigma-Aldrich, USA) was dissolved in 100 mL of deionized water to prepare a 5 mM solution via magnetic stirring for 10 min. Subsequently, 0.6 mL of 30 wt.% hydrogen peroxide (H_2_O_2_, Sigma-Aldrich, USA) was introduced into the solution, which was then stirred for an additional 30 min. Oxalic acid (C_2_H_2_O_4_, 0.645 g, Sigma-Aldrich, USA) was added, and the mixture was stirred for 1 h. Potassium carbonate (K_2_CO_3_, Sigma-Aldrich, USA) was gradually incorporated to adjust the pH of the solution to 10.5. The solution was then stirred in the dark for 5 days to yield the electroplating bath.

### 2.3. The Impedance and Stimulation Performance Test Methods of Electrode

To characterize the electrical and electrical stimulation-related properties of the electrodes, electrochemical impedance spectroscopy (EIS), cyclic voltammetry (CV), and voltage transduction performance were conducted. All tests were performed in a 1× concentration PBS solution (Biosharp Life Sciences, Beijing, China). Impedance and CV scans were performed using the PSTrace4 electrochemical workstation. The tests were conducted in a three-electrode system, with the flexible probe serving as the working electrode, a Pt wire as the counter electrode, and Ag|AgCl as the reference electrode. The impedance test was performed with a scan frequency ranging from 1 to 1000 kHz. For CV scans, the voltage range was set from −0.2 V to 0.8 V, with a scan rate of 50 mV/s.

**Figure 4:**
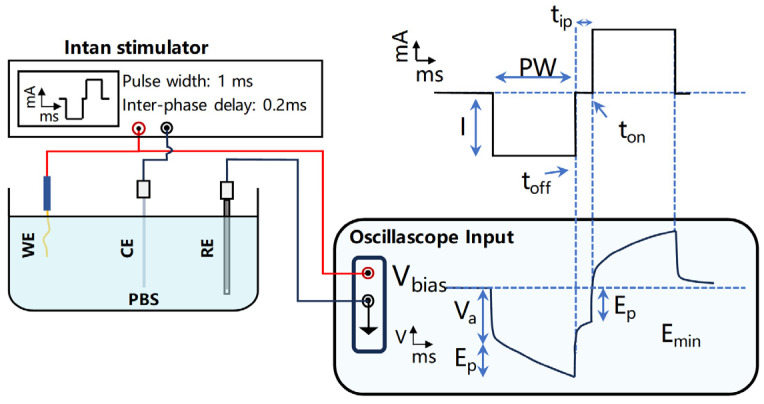
Schematic diagram of the voltage transconductance performance testing apparatus.

The evaluation of voltage transconductance properties was conducted by applying electrical pulses between the flexible probe and a Pt wire, while recording the resultant voltage fluctuations across the flexible probe and an Ag|AgCl reference electrode, as shown in Figure 4. The electrical pulses [20,21] were delivered by an Intan RHS 32-Channel Stim/Recording System (Intan Technologies), with charge-balanced biphasic pulses at a frequency of 50 Hz, a pulse width (PW) of 1 ms, and an interphase delay (t_ip_) of 0.2 ms. The voltage transient curves were captured and documented using a 3 Series MDO Oscilloscope (Tektronix, Inc., Beaverton, OR, USA).

### 2.4. Electrical Recording and Analysis of Neural Activity

The acquisition of murine cerebral electrophysiological signals was executed with precision utilizing the Intan RHS 32-Channel Stim/Recording System. The interfacing of the indwelling flexible probe apparatus with the system was achieved through the integration of an omnetics connector on the PCB. Additionally, the reference electrode was adroitly affixed to the front-end acquisition headstage, ensuring the integrity of signal capture.

The system was linked to the RHS Stim/Recording controller through the Stim SPI interface cable. The mice were allowed to freely move in a spacious 30 × 30 cm black acrylic box for signal collection and analysis. The Intan RHS 32-Channel Stim/Recording System software (Version 3.3.1) provided real-time monitoring of the neural signals. After the collected neural electrical signals were saved, further analysis was conducted using offline sorter to observe the activity of single neurons. The recorded electrical signals in RHS format from Intan were converted to the nex format for analysis in the offline sorter software.

### 2.5. Behavioral Modulation and Analysis in Mice

To ascertain the neuromodulation efficacy of the implanted flexible probe, the investigation involved the surgical implantation of the probe within the bilateral M2 cortical areas of mice [22,23]. The potency of neural stimulation and its consequential influence on mice behavioral responses were quantified through the assessment of pronounced rotational locomotion after the activation of the cerebral regions.

To mitigate the perturbations induced by the ensnarement of cabling from the data acquisition apparatus during the rodents’ rotational activities, the cables were ingeniously retrofitted with a central swiveling pulley system. Throughout the experimental protocol, subjects were accorded unrestrained ambulation within a commodious enclosure, specifically a 30 × 30 cm arena constructed from opaque black acrylic to ensure a controlled environment. The elicitation of electrical stimuli and concurrent signal capture were executed via an Intan recording system, ensuring high-fidelity data acquisition.

The parameters for the stimulation signal were calibrated as follows: a 200 μs cathodal pulse with a charge density of 5 μA (1 nC/phase), succeeded by a 100 μs interphase interval, and culminating in a 400 μs anodal pulse at an amplitude of 2.5 μA, all delivered at a consistent frequency of 100 Hz. The activation of distinct cerebral loci was orchestrated via keyboard commands, facilitating the systematic observation of murine rotational behaviors elicited by the stimulation of specific neural substrates.

**Figure 5:**
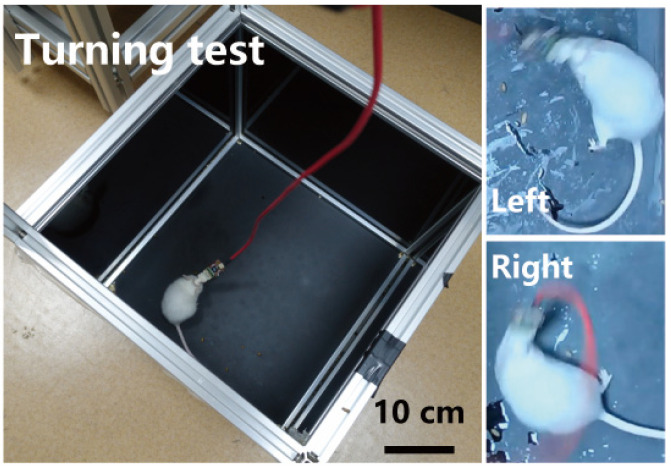
Mouse behavioral modulation experiment device for electrical stimulation. **Left**: Mouse behavior regulation experimental apparatus. **Right**: Close-up images of mouse turning behavior in response to stimulus.

To monitor and quantify the turning behaviors of the mice, a camera was strategically positioned above the black enclosure to capture their activity throughout the stimulation period, as shown in Figure 5. After the recording phase, video analysis was conducted to track the spatial coordinates of the mouse. The resultant data pertaining to the mouse’s positional changes were extracted and employed to construct a detailed profile of the turning behavior elicited by the neural stimulation.

## 3. Results and Discussion

### 3.1. The Design and Morphology of a Flexible Probe

To enable the recording and stimulation control of bilateral brain electrical signals, this study employed a flexible probe design with 32 channels and dual shanks (see Figure 2. The electrode spacing was set at 0.5 mm, with a length of 5 mm, allowing for the flexibility to adjust the depth and spacing of the implantation according to experimental design. The 32 electrode sites were evenly distributed across two shanks, each containing 16 electrode sites configured in a linear array. The distance between electrode points was 75 μm, resulting in a total flexible probe length of 1.125 mm, which can cover the depth of the implanted brain area (M2). The apertures of the electrodes were dimensioned to 15 × 25 μm. The probe utilized a single-layer of polyimide with a thickness of 1.2 μm as the insulation layer, resulting in an overall thickness of 2.5 μm. The bending modulus of the probe was 3.9 × 10^−13^ N·m^2^, which minimized the mechanical mismatch between the probe and the brain. As a result, during long-term implantation, the damage of brain tissue caused by brain micromovements was reduced. The utilization of ball grid array (BGA) packaging at the backend of the device has significantly reduced the overall size of the device, with a pad spacing of 0.6 mm and a total width of only 2.3 mm.

**Figure 6:**
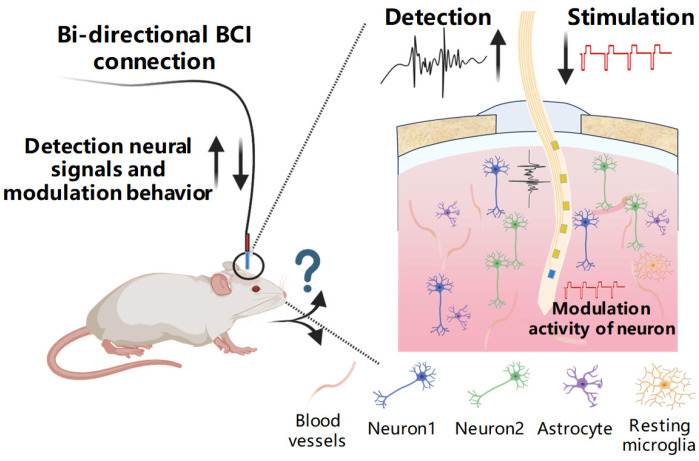
Schematic diagram of the electrical signal recording and movement modulation system for mice implanted with a flexible recording–stimulation bidirectional probe.

To establish the connection between the probe and the Intan RHS 32-Channel Stim/Recording controller, a dedicated PCB was designed. The front end of the PCB utilized a 4 × 8 BGA array with a spacing of 0.6 mm, perfectly matching the pad size of the device. The back end of the PCB employed an omnetics interface to connect with the Intan RHS 32-Channel Stim/Recording Headstages. The overall width of the PCB was a mere 12.45 mm, and the packaged weight of the entire system was only 0.73 ± 0.02 g, the schematic diagram of the overall system connection is shown as in Figure 6.

### 3.2. SEM and Electrochemical Properties of Modified Electrodes

**Figure 7:**
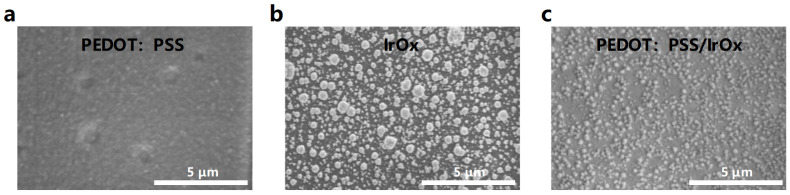
SEM images of electrode surfaces modified by different methods. (**a**–**c**): SEM images of PEDOT: PSS, IrOx, PEDOT: PSS/IrOx respectively.

Scanning electron microscopy (SEM) is a crucial characterization technique for researching the morphology of electrode surface modifications, as shown in Figure 7. SEM analysis has revealed that the surface of the microelectrodes modified with PEDOT:PSS displays a relatively uniform roughness at the microscale. Conversely, the modification with IrO_x_ is characterized by the presence of larger, particle-like nanograins, indicative of a distinct surface topography. The synergistic integration of PEDOT:PSS with IrO_x_ modifications culminates in a more homogenized and fine dispersion of IrOx particulate nanograins upon the consistently roughened PEDOT:PSS substrate. This composite modification strategy results in an optimally textured electrode surface conducive to enhanced electrochemical performance. The resultant surface, upon undergoing composite modification with PEDOT:PSS/IrO_x_, exhibits a markedly uniform morphology alongside an expanded specific surface area. The IrO_x_-modified surface is characterized by the emergence of smaller particles, serving to augment the effective contact area of the electrode. This dual modification not only amplifies the specific surface area but also acts to significantly elevate the electrochemical activity of the electrode, thereby potentially improving its performance in various applications.

**Figure 8:**
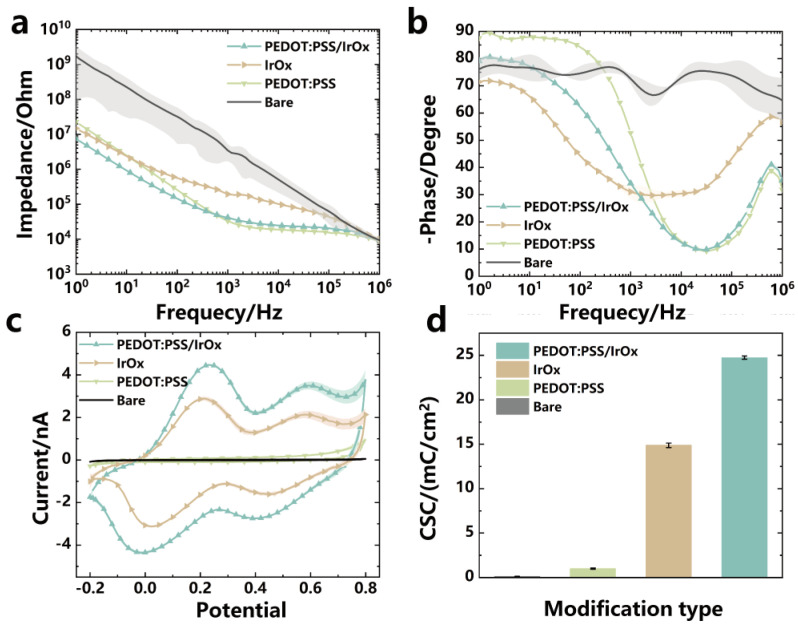
Comparison of electrical performance and charge storage performance tests. (**a**) Impedance curves of electrodes obtained by different modification methods, n = 8. (**b**) Phase curves of electrodes obtained by different modification methods, n = 8. (**c**) CV curves of electrodes obtained by different modification methods, n = 8. (**d**) Comparison of CSC by different modification methods, n = 8.

EIS represents a critical analytical technique for evaluating the impedance characteristics of electrode systems [24]. The minimization of electrode impedance is of paramount importance for enhancing the quality of electrophysiological signal acquisition, as shown in Figure 8a,b. The EIS analysis demonstrated that the unmodified electrode exhibited a relatively high impedance value of 3.47 ± 1.77 MΩ at 1 kHz. Post-modification with PEDOT:PSS, there was a pronounced decrement in impedance, down to 33.17 ± 0.68 kΩ, illustrating a significant enhancement in conductivity. Subsequent modification with IrO_x_ yielded an impedance of 195.22 ± 6.97 kΩ. The incorporation of both PEDOT:PSS and IrO_x_ modifications resulted in a further reduction in impedance, achieving a lower value of 41.88 ± 4.04 kΩ, indicative of a synergistic effect in optimizing the electrode’s impedance characteristics [25,26,27].

In the present investigation, electrodes subjected to material-specific modifications demonstrated discernible disparities in their frequency response profiles. Notably, these modified electrodes manifested distinct impedance behaviors during high-frequency signal transmission and exhibited capacitance features within the low-frequency domain. The phase at 1kHz frequency, the unmodified electrode, the electrode modified with PEDOT:PSS, the electrode modified with IrO_x_, and the electrode modified with PEDOT:PSS/IrO_x_ composite had phase angles of −72.61 ± 1.84°, −52.65 ± 0.24°, −31.05 ± 2.43°, and −34.17 ± 0.42°, respectively. It is worth noting that at 1kHz, the phase of the electrode modified with PEDOT:PSS/IrO_x_ composite was approximately increased 52.37% of the phase of the unmodified electrode. Furthermore, the electrode modified with PEDOT:PSS/IrO_x_ demonstrated good phase stability, which is important for enhancing the electrode’s signal acquisition capability and reducing waveform distortion.

In the performance indices critical to neural microelectrode design, CSC plays an indispensable role. It denotes the total amount of charge that the electrode material can store at its surface or in the vicinity thereof. This corresponds to the maximum amount of charge that can be safely injected or absorbed by the electrode without inducing irreversible chemical reactions outside of the electrolysis window. Electrodes with a high CSC can store and transfer more charge without causing damage to the electrode material and surrounding biological tissues. This facilitates more effective electrical stimulation and reduces the likelihood of irreversible electrochemical reactions that could occur with long-term use, such as corrosion of electrode materials or damage to biological tissues. Therefore, the ideal electrical stimulation electrode possesses a high CSC, enabling it to deliver the necessary current at a lower electrode–tissue interface impedance, thereby enhancing stimulation efficiency. To quantitatively measure CSC, the CV technique is employed. Specifically, the value of CSC is obtained by dividing the area (*Q*) under the CV curve, obtained through integration, by the scan rate (*v*) and the effective surface area (*A*) of the microelectrode. This can be mathematically expressed as:

(1) Q=∫idE

(2) $$CSC=\frac{Q}{\nu A}\;$$

Here, *i* and *E* represent the current and voltage, respectively, in the CV test. *Q* represents the charge corresponding to the closed area enclosed by the CV curve. Meanwhile, *v* and *A* represent the scan rate and the surface area of the electrode in the CV test, respectively. The CSC value obtained through this method reflects the electrode’s charge storage capacity per unit area, which is an important parameter for evaluating its performance in electrical stimulation in neurophysiology.

The surface modification of microelectrodes with different materials has a significant impact on their CSC, as shown in Figure 8c,d. This study revealed that the unmodified electrode had a CSC of 0.14 ± 0.01 mC/cm^2^. After modification with PEDOT:PSS, the CSC increased to 1.00 ± 0.06 mC/cm^2^. Following the modification with IrO_x_, the CSC dramatically increased to 14.88 ± 0.26 mC/cm^2^. Furthermore, when the microelectrode surface was modified with PEDOT:PSS/IrO_x_, the CSC reached 24.75 ± 0.18 mC/cm^2^. From these data, it can be calculated that the CSC of the microelectrode modified with PEDOT:PSS/IrO_x_ was approximately 177.24-fold higher than that of the unmodified electrode. It was also approximately 24.66-fold higher than the electrode modified with PEDOT:PSS alone and approximately 1.66-fold higher compared to the electrode modified with IrO_x_ alone. These results clearly demonstrate the significant enhancement of microelectrode CSC through material modifications, which have the potential to improve the efficiency of charge–discharge processes and reduce the potential for tissue damage in neural electrodes.

### 3.3. The Charge Transfer Properties of the Modified Electrodes

The charge injection capacity (CIC) of an electrode is the parameter for evaluating electrical stimulation electrodes, where the maximum charge injection is defined as the greatest amount of charge that can be transmitted by the electrode without inducing electrochemical reactions. In practical measurements, the standard for testing the maximum charge transfer of an electrode is that the electrode potential does not exceed the reduction potential of water (−0.6 V) during the application of current pulses [28]. If the potential exceeds −0.6 V, electrolysis of water could occur, generating harmful substances that could damage surrounding biological tissues and accelerate electrode degradation.

**Figure 9:**
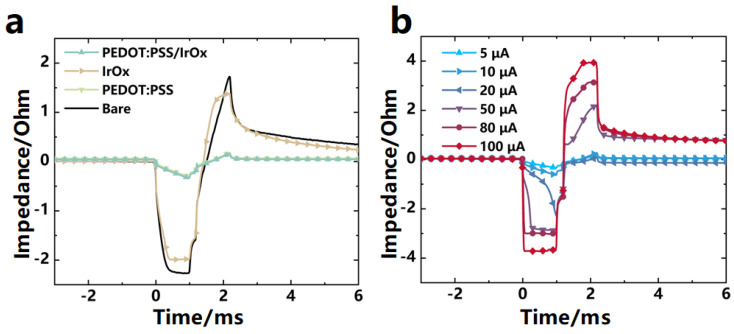
Comparison of voltage transient performance tests. (**a**) Voltage transconductance curves of electrodes obtained by different modification methods, n = 8. (**b**) Voltage transconductance performance of PEDOT:PSS/IrO_x_-modified electrodes under different currents, n = 8.

Figure 9a presents the voltage transient profiles for a variety of electrodes subjected to 5 μA charge-balanced current pulse stimulations. These profiles chart the temporal evolution of the electrode potentials. Occurrences where E_p_ falls below −0.6 V are indicative of impending water electrolysis, whereas E_p_ values exceeding −0.6 V denote operation within the electrochemical safety margin for aqueous environments. The unmodified electrodes exhibited an E_p_ of −1.59 ± 0.01 V, significantly below the reduction potential of water, indicating a poor safety margin during stimulation. Microelectrodes modified with PEDOT:PSS showed an E_p_ of −0.20 ± 0.01 V, well above the reduction potential of water, suggesting a robust charge injection capability and enhanced safety during stimulation. Electrodes modified with IrO_x_ had an E_p_ of −1.54 ± 0.01 V, which is also below the reduction potential of water, indicating that despite the enhanced charge injection ability conferred by the IrO_x_ modification, safety under these stimulation conditions may still be compromised. Microelectrodes that were subjected to a composite modification with PEDOT:PSS/IrO_x_ exhibited an E_p_ of −0.23 ± 0.01 V, surpassing the reduction potential of water, demonstrating that the composite modification effectively improves charge injection capacity while maintaining the safety of stimulation.

The variation in electrode potential with increasing stimulation currents offers additional evidence that the modified electrodes can maintain their potentials within a safe range across a broader spectrum of currents, as illustrated in Figure 9b. This indicates that the modified electrodes can deliver greater charge quantities without exceeding the reduction potential of water, thereby enhancing the safety of the electrical stimulation process.

In summary, modifications with PEDOT:PSS and PEDOT:PSS/IrO_x_ have significantly improved the charge injection capabilities of microelectrodes, reduced peak potentials, and facilitated the maintenance of potentials above the safety threshold over an expanded range of currents, thus enabling safer electrical stimulation. These characterization outcomes are of critical importance for guiding the design of high-performance microelectrodes for bioelectrical stimulation applications.

### 3.4. In Vivo Recording of Neural Electrical Signals by the Implanted Probe

**Figure 10:**
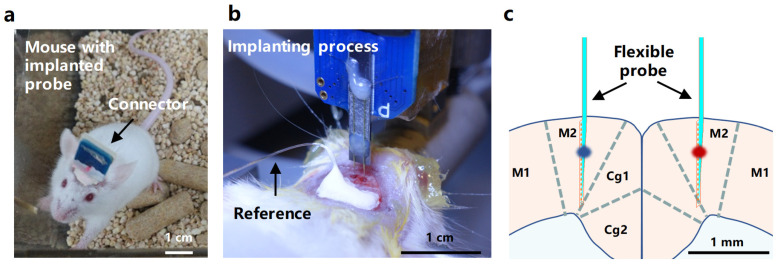
In vivo functional validation of the flexible probe. (**a**) A mouse moving freely after implantation of the flexible probe. (**b**) Photo of the surgical process for implanting a flexible probe in the bilateral M2 region of the mouse. (**c**) Schematic diagram of the position of the 2 shank 32-channel flexible probe implanted in the mouse brain tissue.

The implantation of flexible probes and the recording of electrophysiological signals are crucial techniques in neuroscience research for monitoring and understanding brain electrophysiological activity as shown in Figure 10. The assembly and in vivo implantation methods of flexible probes are shown in Appendix A. Implanting flexible probes into the M2 area of mice allows for the collection of electrical signal data from this specific brain region.

**Figure 11:**
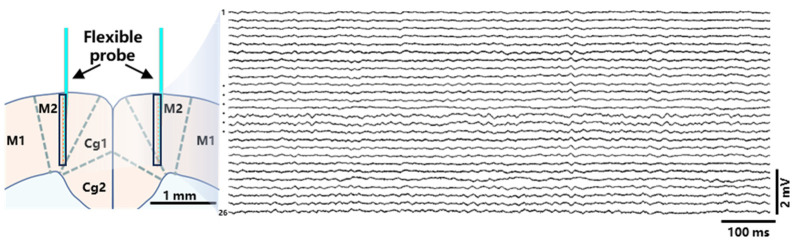
The raw neural signal waveforms recorded after the 32-channel probe with 26 conduction channels was implanted in the M2 region.

The electrophysiological signals recorded in real time using the Intan RHS 32-Channel Stim/Recording System typically consist of components at various frequencies, providing different types of neural activity information. Figure 11 shows the original neural signal recorded by the 32-channel probe implanted in the mouse. After removing the six non-conducting channels, there are a total of 26 available channels.

**Figure 12:**
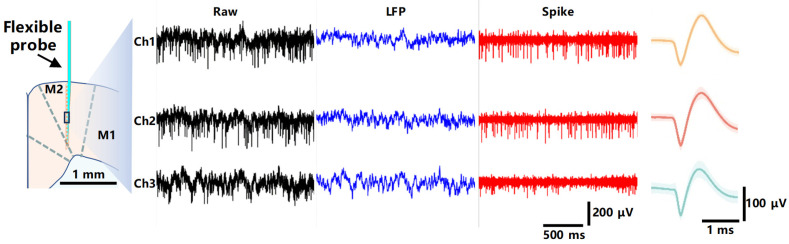
Neural signals recorded in the M2 region by implanted probe at 3 channels with full bandwidth (raw), low-pass (LFP), and high-pass (spike) filtering and corresponding analysis of sorted spike waveforms.

As shown in Figure 12, the signal components consist of high-frequency components (frequency > 250 Hz) and low-frequency components (frequency < 250 Hz). High-frequency components typically include the action potentials of neurons. Action potentials, referred to as “spikes,” are indicative of neural firing, representing the way neurons transmit information through their axons. The recorded spike activity can be used to identify the discharge patterns of individual neurons, including their firing rates and patterns. The high-pass filtered signals enable the extraction of single-neuron discharge waveforms. The SNRs for the waveforms across the three channels are as follows: 8.49, 18.76, 15.82. The low-frequency components encompass LFPs, which are electrical signals generated by the collective activity of groups of neurons. LFPs reflect the synchronous activity of neuronal populations and are often associated with the overall state, functional connectivity, and information processing within neural networks. Signal processing analysis involves the use of corresponding filters. The neural firing signals are analyzed using high-pass filters to extract the high-frequency components. Low-pass filters are employed to filter the signals to analyze LFPs, preserving components below 250 Hz.

The results of the present investigation reveal that the enhanced flexible probe manifest a heightened efficacy in the acquisition of electrophysiological signals. This stride in probe technology lays a robust groundwork for probing the interplay between neural electrical dynamics and animal behavior, thereby advancing the intricate exploration of the underlying mechanisms governing cerebral functionality.

### 3.5. Electrical Stimulation Modulates Mice Behavior

To validate the electrical stimulation performance of the improved flexible probe, this study implanted the probe into the left and right M2 regions of experimental mice. The Intan RHS 32-Channel Stim/Recording controller was used to deliver electrical stimulation to the mice.

**Figure 13:**
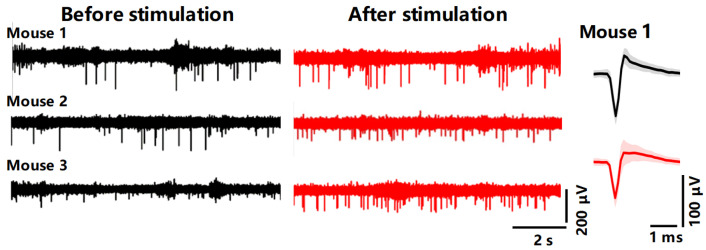
Comparison of high-pass (spike) filtering signals collected before and after stimulation in different mice and comparison of spike waveforms sorted before (black) and after (red) stimulation in Mouse 1.

As depicted in Figure 13, the neural activity recorded by the modified flexible probe in mice is significantly evident before and after electrical stimulation. Both before and after stimulation, distinct neuronal spike potentials can be observed. Furthermore, following the application of electrical stimulation, there is a significant increase in the firing frequency of some neurons while the average peak potential waveform remains stable, reflecting the stability of the electrophysiological recordings.

**Figure 14:**
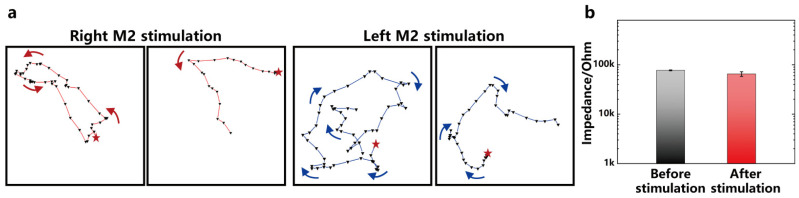
(**a**) Mouse turning trajectory under unilateral M2 region stimulation. Right M2 stimulation causes the mouse to turn left, and left M2 stimulation causes the mouse to turn right. (**b**) Comparison of electrode impedance before and after stimulation, n = 6.

During the experiment, electrical stimulation was applied while the mice were maintained in a quiescent state. As depicted in Figure 14a, after the application of electrical stimulation, the mice swiftly performed unilateral turning behavior, with the asterisk indicating the starting point of the behavior. When electrical stimulation was applied to the right side of the M2 region, the mice quickly turned to the left. Conversely, when electrical stimulation was applied to the left side of the M2 region, the mice turned to the right. This result suggests that the improved flexible probe can effectively control the movement behavior of the mice through electrical stimulation.

Figure 14b illustrates the changes in electrode impedance before and after electrical stimulation. The results demonstrate that the impedance of the electrodes remains stable following electrical stimulation, indicating that the flexible electrodes exhibit excellent stability during both the stimulation processes. This finding highlights the reliable performance of the flexible probe in maintaining stable impedance throughout the experiment.

## 4. Conclusions

This study introduces a novel method of surface electrode modification using PEDOT:PSS/IrO_x_ composite deposition for an ultraflexible neural recording–stimulation bidirectional probe. Leveraging the MEMS surface processing technology, we achieve precise fabrication of the probe, resulting in a probe thickness of only 2.5 μm and a width of 100 μm. The cross-sectional area of the flexible probe is 250 μm, leading to a bending modulus of only 3.9 × 10^−13^ N·m^2^, reducing mechanical mismatch with brain tissue by 35 fold compared to silicon-based electrodes of similar dimensions.

The PEDOT:PSS/IrO_x_ composite modification method significantly enhances impedance and CSC compared to bare and singly modified materials, greatly improving the electrode performance in neural signal recording and stimulation. Implantation experiments in the mouse motor cortex validate the application of the flexible probe for high signal-to-noise ratio signal recording and modulation of mouse motor neural signals, showcasing the extensive potential of these electrodes in neuroscience research and neurodegenerative disease studies.

Moving forward, by defining the unique morphology and functional characteristics of an ultraflexible probe in specific neuroscience research or through further advancements in materials and processing techniques, our proposed PEDOT:PSS/IrO_x_ composite-modified ultraflexible recording–stimulation bidirectional probe will drive significant clinical and research applications in the field of neuroscience.

## Data Availability

The data presented in this study are available upon reasonable request from the corresponding author.

## Appendix A

Assembly and in vivo implantation of a flexible probe: To evaluate the efficacy of a flexible probe for concurrent recording and stimulation after intracorporeal implantation, we proceeded with the insertion of modified electrodes into the encephalic tissue of murine models. To expedite the implantation process of these flexible probe, a robust tungsten wire, measuring 50 μm in diameter and 100 mm in length (Xiamen Honglu Tungsten and Molybdenum Industry Co., Ltd., Xiamen, China), was employed as a rigid shuttle needle, providing indispensable structural support during the delicate insertion procedure.

Prior to assembling the probe, we applied an appropriate amount of Ni etchant to the encapsulated device’s front end to corrode the sacrificial Ni layer, thus separating the flexible probe from the silicon substrate. The corresponding silicon portion of the flexible probe was removed, completing the release of the flexible probe. Next, we proceeded with the assembly of the flexible probe. We fixed two quartz tubes (ID: 100 μm, OD: 150 μm, Shanghai Gutiao Technology, Shanghai, China) at the backside of the device, with a spacing of 1.5 mm matching the actual distance between the left and right brain regions.

The fabrication process entailed threading tungsten wire through quartz tubing, ensuring that a precise length of ~3 mm of wire protruded from the distal end. To facilitate a transient yet secure attachment of the flexible probe to the tungsten wire’s leading edge, we employed a fine brush to apply a 10% polyethylene glycol (PEG) solution with M.W. 100 k or the silk fibroin (wt. 4–7%) solution, which served as a controlled, soluble adhesive.

All animal procedures were carried out at Shanghai Laboratory Animal Research Center, Shanghai, China. All animal-related experiments were approved by the Institutional Animal Care and Use Committee of Shanghai Lab. Animal Research Center (approval number: PA202301002). Male adult mice (ICR, 8 weeks old) were used for these studies. Initially, murine subjects were gently introduced into an induction anesthesia chamber where they were administered a 3% isoflurane–oxygen mixture with a flow rate set at 3 L/min to induce a state of anesthesia. Upon the cessation of voluntary movement, indicating adequate anesthetic depth, the mice were positioned and secured within a stereotaxic apparatus, with precise adjustments made to the ear bars to ensure firm yet gentle cranial stabilization. After immobilization, the isoflurane concentration was judiciously reduced to 1.5% to maintain anesthesia, concomitant with a proportional reduction in the oxygen flow rate, thereby optimizing the anesthetic regimen to safeguard the physiological well-being of the subjects during the ensuing experimental procedures.

To obviate the risk of hypothermia and maintain a consistent core body temperature throughout the surgical intervention, a heating pad (Shenzhen Ruiwode Life Technology Co., Ltd., Shenzhen, China) was positioned beneath the mice. In anticipation of potential ocular damage from the surgical luminescence, a protective layer of erythromycin ophthalmic ointment was applied to the eyes of the mice. Prior to incision, the cranial pelage was excised using a precision electric shaver, ensuring a clear operative field. This was followed by a thorough aseptic preparation of the exposed skin with an iodine solution to minimize infection risks. Subsequently, the scalp was excised to reveal the underlying cranial surface, readying the site for surgical manipulation.

Subsequently, the superficial fascia was excised from the cranial bone, and any residual bone wax was eradicated to ensure an unobstructed surgical site. Utilizing the bregma as an anatomical landmark, the coordinates for probe implantation were delineated as follows: anteroposterior (AP) +1 mm, mediolateral (ML) ± 0.75 mm, and dorsoventral (DV) −2 mm. The reference electrode was slated for implantation at coordinates AP: −1.5 mm, ML: +1.5 mm, DV: −2 mm, as shown in Figure 10c. Upon accurate demarcation of these coordinates, the skull was perforated at the predetermined loci using a high-precision dental drill. This allowed for the precise and minimally invasive insertion of the probe at the targeted intracranial positions.

Initially, a 0.2 mm diameter stainless steel wire was adeptly positioned to serve as the reference electrode and was subsequently anchored in place using dental acrylic cement to ensure its stability, as shown in Figure 10b. Following the successful placement of the flexible probe, PBS was applied to the exposed cranial surface. This application was intended to facilitate the full dissolution of the PEG matrix, a process allowed to proceed unhindered for a duration of 10 min. To culminate the procedure, the supporting tungsten wire was delicately extracted, signaling the completion of the flexible probe implantation.
